# SPA-IoT with MCSV-CNN: a novel IoT-enabled method for robust pre-ictal seizure prediction

**DOI:** 10.1186/s12911-025-03191-5

**Published:** 2025-09-29

**Authors:** Dhanalekshmi Prasad Yedurkar, Shilpa P. Metkar, Thompson Stephan, Vijay Mohan, Saurabh Agarwal

**Affiliations:** 1 School of Computing, MIT Art Design & Technology University, Pune, India; 2 Department of Electronics & Telecommunication and Engineering, COEP Technological University, Pune, India; 3 Thumbay College of Management and AI in Healthcare, Gulf Medical University, Ajman, United Arab Emirates; 4 Department of Mechatronics, Manipal Institute of Technology, Manipal Academy of Higher Education, Manipal, Karnataka 576104 India; 5 School of Computer Science and Engineering, Yeungnam University, Gyeongsan, 38541 Republic of Korea

**Keywords:** Seizure prediction, EEG, IoT, Deep learning, Multiresolution critical spectral verge

## Abstract

This paper introduces a new approach to real-time epileptic seizure prediction using a lightweight Convolutional Neural Network (CNN) architecture and multiresolution feature extraction from electroencephalogram (EEG) recordings. Multiresolution Critical Spectral Verge CNN (MCSV-CNN), the suggested model, is best suited for use in wearable technology that is connected to the Internet of Things (IoT). The software module uses pre-ictal and inter-ictal EEG segments to forecast seizures early, and the signal acquisition module collects EEG data. Multiscale frequency analysis and spatial feature learning are combined in the MCSV-CNN architecture to capture minute signal changes that precede seizures. Both actual clinical EEG recordings and the Temple University Hospital EEG Seizure Corpus (TUH-EEG) were evaluated. Predicting has been performed using a 5-minute pre-ictal window and a 10-minute seizure occurrence prediction (SOP) horizon. The approach proposed outperformed a number of existing CNN-based seizure prediction techniques with an average prediction accuracy of 99.5%, sensitivity of 98.3%, false prediction rate of 0.045, and a high Area Under the Curve (AUC). These findings show that MCSV-CNN has the potential to be a dependable, real-time seizure prediction tool that could be used practically in wearable medical technology. The prediction accuracy and lightweight architecture of the technology point to its potential application in early clinical intervention and ongoing at-home monitoring.

## Introduction

**Fig. 1:**
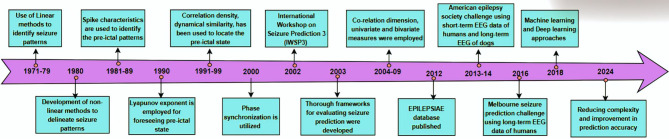
Summary of research in the direction of prediction of epileptic seizure

**Fig. 2:**
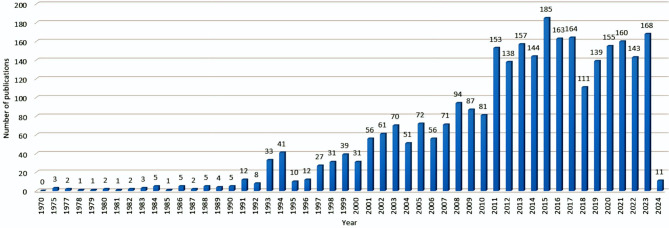
An overview of the quantitative comparisons presented in earlier studies

Health and wellness surveillance systems that operate in the realm of IoT make it possible to monitor individuals continuously, especially in the case of chronic conditions and long-term surveillance applications like epileptic seizures (ES)  [1], [2]. IoT, through its multiple sensors and networks, plays a crucial role in creating a direct line of communication of medical information between medical sensors and devices attached to patients and medical personnel. In doing so, it provides continuous medical monitoring of very significant seizure data of patients like the pre-ictal (PIL) state  [3], [4]. Information such as this is extremely useful in the remote prediction of the occurrence of seizures, thus facilitating immediate and pre-emptive assistance. Epilepsy is known to be a lingering neurological disease that afflicts nearly 70 million human beings the world over  [5]. Seizure prediction (SP) is performed by identifying the pre-seizure (PIL) region, a few minutes to seconds before the seizure onset, from the EEG recordings. Predicting ES is crucial for several reasons, and it can greatly enhance the lives of people with epilepsy [6]. Here are some key reasons why predicting ES is essential: i. early intervention and treatment planning; ii. Enhancing safety; iii. Improving quality of life; iv. Reducing side effects of medication; v. Optimizing healthcare resource utilization; vi. Enabling personalized treatment; vii. Facilitating research and understanding; and viii. Empowering epileptic individuals. Although there have been major advances in SP, predicting seizures with high accuracy remains a complex challenge, and ongoing research is essential to improve prediction capabilities. Figs. 1 and 2 summarize prior research in this direction.

Currently, various algorithms are used to predict the epileptic seizure state. The most prevalent types of algorithms include time domain, frequency domain, a combination of frequency-time domain, and non-linear approaches. Out of all the methods that are used, the frequency spectral analysis has been fairly successful  [7]. Correlation dimension, Lyapunov exponent, and approximate entropy are techniques that have been employed in feature analysis of the prediction of ES  [8]. Besides the single-channel analysis of EEG, current research has begun to concentrate on the inter-relationship that exists between multichannel EEG seizure signals  [6], [9].

Deep learning (DL) algorithms are also very useful in the prediction of ES as they are able to identify PIL EEG segments. One type of DL is the Convolutional Neural Network (CNN) [10]. According to research by  [11], CNN time domain analysis is used to operate a fully portable, wireless, flexible scalp electronic system in the occipital lobe. Here, a high information transfer rate has been achieved by a two-channel scalp electronic system with six human participants, enabling real-time EEG categorization. Truong et al. proposed a scheme based on CNN. The input to this CNN is the extricated short-time Fourier transform (STFT) time-frequency map for the identification of PIL as well as inter-ictal (IIL) stages  [12]. Based on STFT CNN, uses 2D CNN to classify EEG segments after extracting spectrograms from them. Despite their effectiveness, they are computationally demanding and necessitate precise window selection. Wavelet-based CNNs provide better temporal resolution; however, they are often patient-specific and susceptible to interference  [13].

Khan et al. proposed an epileptic prediction technique based on wavelet transform and CNN, utilizing scalp EEG electrodes  [13]. Ozcan et al. predicted seizures by using spectral band power, statistical moment, and Hjorth parameters as input to a 3D-CNN model, which had a multi-frame  [14]. Zhang et al. were authors of an innovative solution for predicting ES, making use of a shared spatial pattern along with CNN to enhance the total accuracy even as the technique reduces the time taken for training  [15]. Additionally, research has been undertaken to develop wearable EEG devices  [16],   [17],   [18],   [19]. The following observations are presented in regard to the literature review: There is little proof that seizure prediction algorithms are clinically applicable, despite the fact that many of them have been developed and tested on benchmark datasets. The majority of models undergo retrospective testing with carefully labeled data under controlled conditions, which might not accurately reflect the unpredictability and diversity of actual clinical settings. Furthermore, only a small number of these algorithms have been used in continuous, real-time monitoring scenarios with wearable EEG sensors or verified in prospective clinical studies. Their current usefulness in practice is limited by this lack of clinical validation, which emphasizes the need for methods that are reliable, understandable, and appropriate for inclusion into real-time healthcare systems. This is a significant drawback in the domain of predicting seizures. Both wavelet and STFT-based CNN seizure prediction techniques frequently have low feasibility across data, substantial processing rates, and sensitivity to artifacts. Moreover, there is also much room for improvement in the development of enhanced DL-based classifiers. Besides, the most crucial area that is missed in the current studies is developing an integrated and automated SP system. Such a system can assist with the prediction, diagnosis, and analysis of an oncoming seizure. Hence, there is a need to focus on developing wearable devices that are both handy and compatible with automated systems via the IoT. Multiresolution critical spectral verge (MCSV)-CNN is a lightweight deep learning model that makes use of multi-channel statistical variability data in order to overcome these drawbacks.

While prior work has demonstrated the feasibility of using DL methods, such as STFT-based CNN and wavelet-based CNN, for seizure prediction from EEG signals, these approaches still face several critical limitations. Firstly, fixed-resolution transforms like STFT struggle to effectively capture the nonstationary and multiscale nature of pre-ictal EEG dynamics. Although wavelet transforms offer multiresolution capabilities, many implementations use hand-crafted or rigid decomposition schemes, which may not generalize to patients or types of seizures. Secondly, existing models often prioritize accuracy in offline settings, neglecting important constraints such as real-time processing, energy efficiency, and memory footprint, all of which are essential for on-device inference in wearable IoT systems. Few studies rigorously evaluate whether such models can operate in real-time, particularly in edge-computing scenarios. Third, many methods rely on black-box deep networks without incorporating physiologically meaningful features or robust artifact filtering, which limits clinical interpretability and can degrade performance in noisy real-world EEG. Taken together, these gaps highlight the need for a seizure prediction framework that: a) captures EEG information at multiple temporal and frequency scales, b) supports efficient, real-time deployment on wearable or mobile platforms, and c) enhances interpretability and robustness to artifacts. In particular, sensor placement and attack problems introduce spatial and spectral inconsistencies, which degrade the reliability of prediction systems. These existing models lack mechanisms to extract robust features across multiple time–frequency scales adaptively and are vulnerable to adversarial signals or signal misplacement. To address these challenges, we propose the MCSV-CNN architecture, which combines Multiresolution convolutional subbands and voting strategies to ensure robustness to Seizure Prediction Accessment (SPA)-induced variations, improved generalization across sensor placements, and better resilience to low-SNR or corrupted data segments. In response, this work proposes an MCSV-CNN, which integrates adaptive multiresolution analysis with sub-band energy encoding to reflect the EEG signal structure better while maintaining deployment feasibility. This architecture aims to bridge the gap between theoretical prediction performance and real-world clinical applicability.

The above-mentioned literature survey is ample motivation for the authors to come up with a proposal namely, the SPA-IoT system, with the following main objectives: i) To propose a DL-based SPA system using an EEG signal and classifying it into PIL and IIL states; ii) To come up with an automated IoT based SPA system for real-time analysis; and iii) To improve the false prediction rate (FPR), sensitivity (SEN), and accuracy (ACC) of the proposed SPA-IoT system.

An enhanced aspect of the presented investigation is the prediction of EEG seizures using a novel method known as MCSV in conjunction with a CNN methodology known as MCSV-CNN for an integrated IoT system. Since wavelet-based multiresolution analysis can record brief, nonstationary events across several frequency bands, it is especially well-suited for diagnosing EEG seizures. Wavelets offer adjustable time-frequency resolution, providing high frequency resolution for gradual drifts and high temporal accuracy for fast oscillations, in contrast to the fixed window limits provided by STFT. DL models like CNN can use rich multiscale patterns that indicate pre-ictal activity by converting EEG signals into wavelet scalograms. This improves predicted accuracy and generalization across patients. By extracting the PIL zone from the recordings of a multichannel EEG signal for each segment, the experimental findings demonstrate that this method is capable of predicting ES self-sufficiently. In doing so, it will develop the capability to handle and analyze huge volumes of seizure information. MCSV-CNN will do this by utilizing the MCSV feature that employs a bio-inspired algorithm (BI), like the flower pollination method (FPM)  [20]. Additionally, FPM aids in pinpointing the precise location where ES occurs by performing local and global pollination schemes. Hence, FPM is chosen for the proposed SPA unit. The technique proposed in this research has been tested against data from a publicly available database. It has also been verified against an epileptic signal that was recorded in a local hospital in real time. In accordance with the proposed MCSV-CNN IoT approach, the SPA technique seeks to assist physicians and medical personnel by providing precise diagnoses and offering appropriate therapy, as well as to prevent seizures in epileptic patients by means of timely prediction. While the proposed MCSV-CNN shares structural similarities with the ensemble and multi-path CNN reported in prior literature, its novelty lies in the integration of multiresolution sub-band decomposition directly into the learning pipeline. Unlike conventional CNNs that operate on raw or single-resolution EEG inputs, MCSV-CNN processes multiple frequency-specific representations in parallel, each learned through a dedicated convolutional stream. These frequency bands are not manually engineered but are derived using adaptive multiresolution filtering, allowing the model to learn frequency-dependent patterns more effectively. A majority-voting fusion layer aggregates outputs across these parallel streams, enhancing robustness to noise and inter-subject variability. Additionally, the model is optimized for low-latency inference and lightweight deployment on edge IoT devices, which is rarely addressed in prior seizure prediction CNN architectures.

The following are significant contributions of this research work: i)Designing and developing the novel MCSV-CNN technique to predict seizures based on the EEG data that is obtained, and classifying it into PIL and IIL phases, ii)Demonstrating that it is possible to implement the proposed SPA-IoT technique in real-time, making use of EEG sensors, the internet, as well as a Raspberry Pi, iii)Demonstrating a distinct enhancement in the performance of measures used to predict seizures, like Area Under Curve (AUC), ACC, and also FPR for training and testing data taken from an EEG seizure signal, as compared to the latest techniques used in this field. The suggested prediction method can be a patient-specific seizure warning system that can be designed to be simple to wear and that records continuously in real time. The suggested approach significantly lowers the fatality rate of ES, particularly in low-resource environments where access to both knowledge and treatment is limited. Sect. 2 explains the methodology of the suggested epileptic SP. Sect. 3 is dedicated to analyzing and exploring each segment of the multichannel EEG signal prediction results. Sect. 4 discusses the outcomes of such predictions and compares the MCSV scheme with other SP techniques. Sect. 5 outlines the general conclusion, highlighting the broader implications of the work.

## Methodology

### Problem formulation

Let $X(t) \in \mathbb{R}^{C \times T}$ denote a multichannel EEG segment of duration *T* recorded across *C* electrodes. Each segment is labeled as $y \in \{0, 1\}$, where *y* = 1 represents a preictal window (that is, before a seizure) and *y* = 0 denotes an interictal state (nonseizure).

The goal is to learn a function $f_\theta: \mathbb{R}^{C \times T} \rightarrow \{0,1\}$ such that the predicted label $\hat{y} = f_\theta(X(t))$ approximates the true seizure state:

$${\hat{y} = f_\theta(X(t)) \approx y}$$

The model is trained to:

i. Accurately identify pre-ictal windows within a specified Seizure Prediction Horizon (SPH);
ii. Minimize false positives per hour (FPR/h); and
iii. Generalize across multiple patients and recording conditions.

### Proposed SPA-IoT enabled EEG telemetry framework

**Fig. 3:**
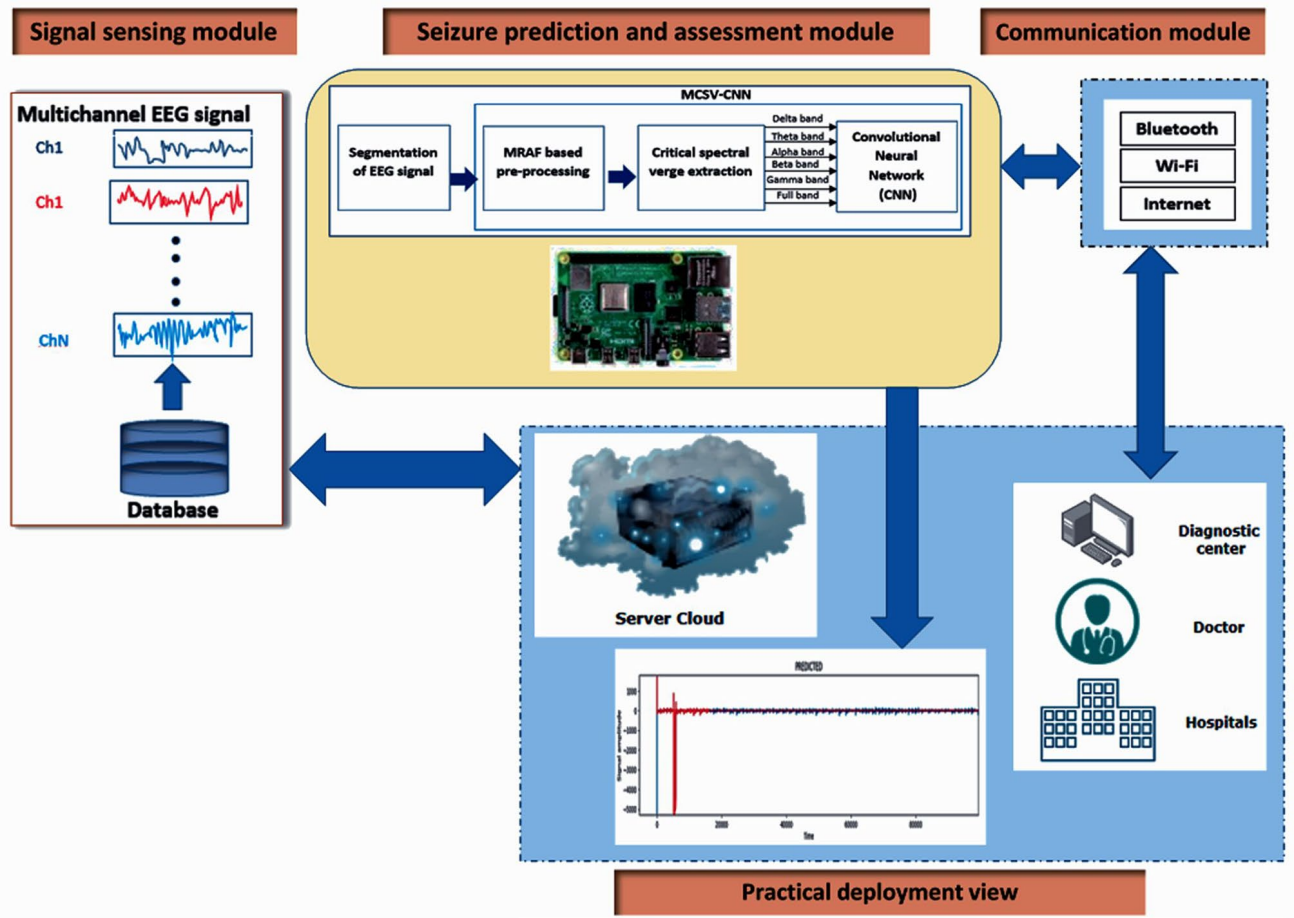
Proposed SPA-IoT framework using MCSV-CNN method for epileptic SP. A high-level overview of the proposed system for predicting pre-ictal seizure using multiresolution EEG analysis and a lightweight CNN model

The SPA-IoT framework is made up of three units: i) the EEG signal sensing unit, ii) the SPA unit, and iii) data analysis of PIL and IIL zones in conjunction with the transmission unit. The SPA-IoT framework is presented in Fig. 3. This research primarily focuses on designing and implementing a real-time EEG SPA prototype that makes use of the MCSV-CNN technique and validates the effectiveness of the suggested SPA-IoT framework in real-time analysis. To begin with, in the suggested MCSV-CNN approach, preprocessing of the multichannel epileptic EEG information is undertaken for each segment, making use of the multiresolution adaptive filtering (MRAF) approach  [21]. Next, an optimized CSV feature is extracted using FPM. The features that are extracted are a representation of EEG frequency bands such as beta (0.5–4 Hz), theta (4–8 Hz), alpha (8–13 Hz), delta (13–30 Hz), gamma (30–100 Hz), and full bands (0.5–100 Hz or up to 250 Hz). Finally, for every segment, the frequency data for consecutive segments is sent over to CNN so that seizures, if any are present, can be predicted.

### Signal sensing modules

A standard dataset and a clinical database were utilized to acquire the epileptic seizure database used in this work.

#### Standard dataset: TUH-EEG

The standard dataset, TUH-EEG, utilized for analyzing the proposed new method is made up of records of hundreds of patients who have had medically complex partial seizures procured from Temple University Hospital Electroencephalography Corpus (TUG-EEG)  [22]. The TUH-EEG includes recordings of 21-channel EEG signals. The recordings also include annotations and are sampled at a frequency that varies between 250 and 400 Hz with a resolution of 16 bits per sample. The data is stored in EDF+, a common electrophysiological signal storage format. The annotations in this version of the database are mostly of two kinds: i) giving the start and end times for seizure events, also including within themselves detailed information about the event, such as channel label and seizure category; ii) providing the classification of the signal, either as normal or abnormal. These are the label files, which provide an in-depth breakdown of every event, thus specifying what takes place within an individual channel or groups of channels. Further categorization and labeling will be essential to the researcher for data analysis in seeking to identify trends and develop predictive models of seizure events. The TUH-EEG dataset was selected due to two key factors: the availability of markings indicating the beginning and end of seizures, as well as information regarding the channel label. Since channel labels are crucial for pinpointing the seizure site on the scalp, they are unavailable from other standard datasets.

#### Clinical dataset

A hospital near the city of Pune, India, provided the second dataset that helped test and validate the suggested methodology. The recording device consists of 21 channels, sampled at 250 Hz, with electrodes positioned according to the international 10–20 electrode placement standard. The sensing unit includes built-in hardware for initial filtering (0.5–60 Hz bandpass) and artifact suppression, and transmits data via Bluetooth for real-time or offline analysis. The unit’s resolution was 16-bit, and it was capable of detecting subtle pre-ictal variations with minimal latency, making it suitable for real-time seizure prediction. Each patient underwent recording sessions, resulting in a dataset that included both interictal and pre-ictal segments annotated by experienced clinicians. Informed consent was obtained from all individual participants included in the study. This dataset was used after obtaining permission from the ethical committee. In the case of EEG recordings that varied from 1 to 8 hours and were unipolar, from 5 distinct patients.

### Seizure prediction and assessment

In this research, preprocessing is undertaken for the multichannel EEG information for every segment, making use of MRAF  [21]. Next, the MCSV feature is extracted using FPM. For each of the EEG signal’s frequency bands, including theta, alpha, delta, beta, and gamma, the proposed MCSV technique is applied. In the end, the feature set that is thus obtained is fed as input to a CNN model. For this research, 05 minutes prior to the onset of a seizure was selected as the PIL period.

The requirement to balance early detection with effective response time in real-world clinical and wearable IoT uses led to the choice of a 5-minute PIL before seizure onset. While longer windows, such as the 20-minute preictal window used in   [6], will give longer lead periods, they frequently devote a large amount of time to post-processing and preventative actions instead of active forecasting. On the other hand, this study employs multiresolution EEG characteristics for real-time prediction during the 5-minute timeframe, which allows for low-latency replies appropriate for wearable deployment. This period is also in line with results from several recent studies that show the crucial preictal brain dynamics typically show up most strongly in the last few minutes prior to seizure onset. Therefore, the selected window is not only adequate for accurate seizure prediction but also optimally suitable for integration into time-sensitive IoT-based systems.

#### Preprocessing of EEG signals

The multichannel EEG information that is input is divided into segments by using a sliding, non-overlapping window technique  [23]. The sliding window ensures the continuity of the input data. However, it can also result in redundancy of information. This work examined three different window sizes - two seconds, four seconds, and five seconds—analyzing their impact on the outcome of SP to explore the effect of varying non-overlapping window lengths. This analysis revealed that using various segment sizes, with or without segment overlap, had no discernible impact on the epileptic recognition outcomes. Nonetheless, the EEG segment ought not to be so tiny that it takes too long to analyze. Also, the minimum frequency that is procured from the EEG signal is 0.5 Hz  [10],   [19]. Hence, in order to handle the lowest frequency information and on the basis of the pre-experiment, the authors chose a sliding time window of 1040 points for the EEG signal, or 4 seconds with no overlaps.

Details of the EEG signal are frequently impacted by the physiological artifacts present  [24]. Since physiological abnormalities can mask or imitate pre-ictal signs, they provide a significant challenge for EEG-based seizure identification. Reduced model dependability results from these nonstationary phenomena, which frequently overlap with the frequency bands of interest. Therefore, minimizing artifacts that interfere with the precise identification of epileptogenic regions becomes crucial. The current research uses MRAF, which combines scale-wise artifact suppression with wavelet-based decomposition, to address this. This method increases the robustness of downstream seizure prediction models by selectively attenuating artifact-dominated components while maintaining diagnostically significant characteristics  [21].

The first step is the decomposition of the input EEG data into various frequency levels by employing the Discrete Wavelet Transform (DWT). Then, the wavelet coefficients at high frequencies are subjected to soft thresholding that has been procured. Soft thresholding is expressed in Eq. (1) as follows  [25]:

1 $$Z_{l}={\sigma _{l}\sqrt{2 log (P)}}$$

The DWT decomposition level is *l*, *σ*_*l*_ is the input signal’s standard deviation, and *Z*_*l*_ is the threshold value. P is the EEG sample. The MRAF technique is subsequently employed to produce preprocessed data for the seizure region. The data generated by this process is represented by the following MRAF expression in Eq. (2)  [25]:

2 $$D_o(u)_{pr(b)}= \sum_{i=0}^{s-1}{w_i(u) d_i(u-j)}$$

Here, $D_o(u)_{pr(b)}$ represents the output of the MRAF obtained for each band *b*. At the output stage, theta, delta, alpha, gamma, and beta are incorporated, each corresponding to distinct frequency resolutions, thereby achieving multiresolution filtering. The term $w_i(u)$ signifies the weight associated with the MRAF scheme and reflects the adaptive filter’s updated components. Meanwhile, ${d_i(u-j)}$ denotes the most recent updates applied to the adaptive filter.

#### Critical spectral verge computation

The EEG data were preprocessed using the MRAF technique, and feature extraction was performed for every EEG signal segment using the proposed MCSV technique. The proposed MCSV technique is applied to all frequency bands of multichannel EEG data, including theta, alpha, delta, beta, and gamma. The features have been extracted using the MCSV technique to capture the frequency details in the EEG data and highlight the seizure-related information, especially in the high-frequency regions. Feature extraction starts by calculating the average power spectral density for each of the mentioned frequency bands. PSD is computed for each segment in these bands. Then, it identifies the point where the PSD of a segment exceeds the mean PSD of its frequency band. The peak point is called the Spectral Verge (SV). Because this region contains more noise and artifacts, low-frequency segments may distract from the most critical seizure information. The identified SV is further enhanced through the FPM to find an accurate seizure detection. This acts as a seed point, taking the beginning and optimizing it through FPM via Lévy flight. The Critical SV is the resulting optimised point, which is more accurate and reliable for seizure detection in EEG data.

To simplify, let’s call the filtered output per band, $D_o(u)$, as *d*(*u*). We split *d*(*u*) into segments for extracting features and refer to it simply as *d*, dropping the index *u*. The segments of the signal *d* are defined as follows in Eq. (3): Let $d = \{d_1, d_2, \ldots, d_k\}$, where *k* is the segment index.

3 $${k}= \frac {U} {f_s}$$

Here, *k* is the number of EEG segments, *f*_*s*_ is the sampling frequency, and *U* represents the total number of segments.

The filtered signal can be divided uniformly in each band, and this method is applied across each frequency band. The average spectral power (SP) is calculated for all. The SP is evenly dispersed in the high-frequency range, particularly in segments with epileptic data.

Denote the power spectrum of *d*_*k*_ for *b* as $YS_{k_b}$. The set ${YS_k}$ can be written as $Y{S_k} = Y{S_{{k_b}}}(0)$, $Y{S_{{k_b}}}(1), \ldots ,Y{S_{{k_b}}}(m)$, where *m* ranges from 0 to *f*_*s*_. Following this, we compute the average SP.

We are now focused on identifying a frequency point *m* within the range of 0 to $f_s Hz$ that fulfils the requirements listed below: Requirement 1: If the set of frequencies *m*_*s*_ is such that:

4 $$m_s = \{m \mid m \in YS_{k_b}(m) > YS_{\text{avg}}\}$$

Requirement 2: If the SV point is $\max(m_s)$, then:

5 $$\text{SV} = \max(m_s)$$

The SV point computed above has been taken into account as a requirement for the classification of seizure activities. However, since frequency variations of the seizure zones are specific to each patient, there is a possibility of misclassification due to misleading frequency points in certain segments. The authors have chosen the FPM technique as a swarm-based intelligence method for optimization that has been motivated by the way that blooming plants behave during pollination. To address the optimization issue, the FPM algorithm requires the initialization of both the objective function *f*(*d*) and the initial solution. Eqs (4) and (5) have been combined as the objective function, and it can be expressed as shown below in Eq. (6):

6 $$\max \{m \mid m \in YS_{k_b}(m) > YS_{\text{avg}}\}$$

Therefore, the objective function yields the initial solution, which is the best input for the first iteration. The next task is to optimise the obtained initial solution to achieve the ideal fit using the FPM technique. FPM is based on the concept of the abiotic process of pollination, which can be either local or global, to search for the ideal match. To highlight the differences between global and local seizure abnormalities, the FPM algorithm performs repeat passes through both, virtually optimizing the solution for accurate identification of segments with seizure information using the pollination algorithm.

The following Eq. (7) is the definition of the best-suited solution:

7 $$S_i = \text{fit}^*(f_{si})$$

Here, the decision variables vary within the range from 0 to *f*_*s*_, and $fit^*$ denotes the optimal fitness. Consequently, the solution derived will range from the minimum frequency limit $(0 Hz)$ to the maximum frequency limit $(f_s)$. Reaching the optimal fit requires a consistent solution that surpasses all other possible solutions or shows the most frequent occurrence in solution generation. The population function map is defined as given by Eq. (8) below for reaching the ideal solution:

8 $$f: fit^* \rightarrow s_i $$

where the set of solutions obtained from the optimal map for iteration is denoted by *S*_*i*_. At this stage, FPM can be applied to the population that has previously obtained the solution to achieve the ideal solution. In the end, the optimal solution is identified as the CSV, which is refined from all available solutions on the map. Presently, the CSV for each of the other EEG segments can be similarly procured and represented by $\{CSV_1, CSV_2, \dots, CSV_n\}$. The CSV for each of the bands *(α)*, *(β)*, *(γ)*, *(θ)*, *(δ)*, and full band (*fb*) is acquired similarly. Eq. (9) below represents the feature map that has been generated:

9 $$\begin{vmatrix} CSV_{\alpha1},CSV_{\alpha2},...,{CSV_{\alpha k}}\\CSV_{\beta1},{CSV_\beta2},...,{CSV_{\beta k}}\\CSV_{\gamma1}, {CSV_\gamma2},..., {CSV_{\gamma k}}\\CSV_{\theta1}, {CSV_\theta2},...,{CSV_{\theta k}}\\CSV_{\delta1}, {CSV_\delta2},...,{CSV_{\delta k}}\\CSV_{fb1},CSV_{fb2},...,{CSV_{fbk}} \end{vmatrix}$$

**Fig. 4:**
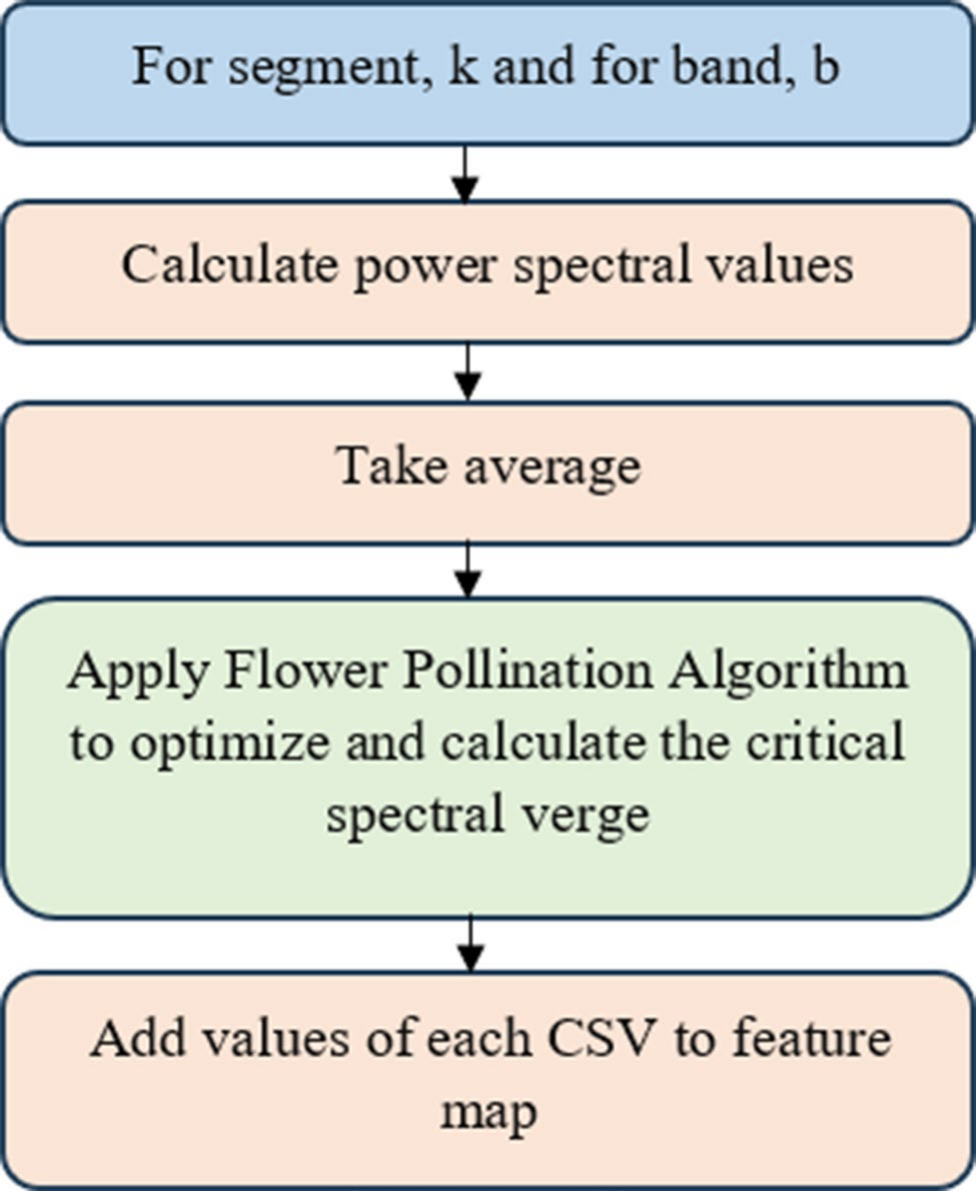
Methodology for the CSV feature computing process flow. A process flow for computing sub-band EEG features using multiresolution decomposition for input into the MCSV-CNN model

The CSV serves as the ideal map, showing the signal connections among EEG segments from 1 to *k*, as well as every frequency band that ranges from alpha to full band, and the channels of the EEG signal. This work proposes a method to compute the optimal feature band based on the CSV. Fig.  4 shows the approach for the CSV feature computation.

#### Convolution neural network

In the last few years, CNN has significantly evolved in the domain of neuro-computing. This is because CNN has many features that give it an advantage, such as feature selection, weight sharing, automatic feature extraction, and so on. As per the AlexNet network, for this study, a six-layer CNN is utilized in order to select features and also for classification  [26]. The suggested CNN model is initialized with a convolutional layer (CL), which uses eight kernels of size 3 × 3with a stride of 1 × 1to filter the frequency feature maps across channels. The second is a CL, taking the output of the first with 16 kernels, while the kernel size is 5 × 5, and the stride is 1x3. Connected after the second in the series, the architecture of the third CL contains 32 kernels in each dimension, kernel size 3 × 3with a stride of 1x3. Finally, max-pooling layers are provided after both the first and second convolution layers. Their pool size is chosen to be 2 × 2with a stride of 2x2.

The ReLU introduces non-linearity in the processing and is used throughout the CLs. Following the convolutional stack, three fully connected layers play a vital role in distinguishing between the PIL signal and the IIL signal. The final output of the fully connected layer goes through a softmax activation function, which produces the probability of being in either the PIL or IIL state. Dropout layers with a rate of 0.5 are added after the first two fully connected layers to avoid overfitting. It processes data in batches of 100, uses categorical cross-entropy for loss, and employs Adaptive Moment Estimation with a 0.001 learning rate for optimization  [27]. Table 1 highlights the layer-wise architecture of the MCSV-CNN.

**Table 1:** Layer-wise architecture of the MCSV-CNN

Layer	Type	Kernel size	Filters	Stride	Activation function
Input	EEG feature maps	-	-	-	-
Conv1	Conv2D	3 ×3	8	1 ×1	ReLU
MaxPool1	MaxPooling2D	2 ×2	-	2 ×2	-
Conv2	Conv2D	5 ×5	16	1 ×3	ReLU
MaxPool2	MaxPooling2D	2 ×2	-	2 ×2	-
Conv3	Conv2D	3 ×3	32	1 ×3	ReLU
Flatten	Flatten	-	-	-	-
Dense1	Fully Connected	-	256	-	ReLU + Dropout (0.5)
Dense2	Fully Connected	-	128	-	ReLU + Dropout (0.5)
Dense3	Fully Connected	-	2	-	Softmax

### Hyperparameter selection

The design of the MCSV-CNN architecture was guided by domain knowledge in EEG signal processing, empirical experimentation, and considerations for deployment on resource-constrained wearable devices.

i. **Kernel size:** Small kernel sizes such as $3\times3$ are effective for capturing local spatial and temporal features, particularly transient patterns found in EEG pre-ictal signals. In the second CL, a kernel $5\times5$ was used to allow the model to integrate slightly more contextual information after initial feature detection.
ii. **Stride:** The asymmetric stride in the second and third CLs ($1\times3$) was selected to prioritize temporal abstraction while preserving the spatial resolution across channels. This allows for better alignment with time-sensitive seizure precursors.
iii. **Number of filters:** A gradually increasing number of filters across layers enables the extraction of increasingly complex patterns. Keeping filter counts relatively low, compared to a very deep CNN, maintains computational efficiency, suitable for wearable implementation.
iv. **Pooling parameters:** Max pooling is applied after the first two CLs to reduce dimensionality while retaining dominant activations. The $2\times2$ pool size with matching stride downscales the input, limiting overfitting and accelerating training.
v. **Dropout:** Dropout layers are inserted after the first two fully connected layers to prevent overfitting, particularly important given the limited availability of seizure data. A dropout rate of 0.5 is commonly used in biomedical CNN applications and was empirically found to provide stable validation performance.
vi. **Batch size:** A batch size of 100 balances training stability and memory efficiency. Smaller batches led to noisier gradients, while larger batches showed diminished generalization.
vii. **Optimize:** Adam was selected for its adaptive learning capabilities and proven performance in non-stationary EEG data settings. A learning rate of 0.001 provided fast convergence without instability.
viii. **Activation function:** ReLU activation was used in all hidden layers due to its simplicity and effectiveness in avoiding vanishing gradient issues. The final layer uses a softmax activation to produce a normalized probability distribution over the PIL and IIL classes.

These choices were supported by preliminary experiments and align with standard practices in EEG-based DL systems. Future work may explore automated hyperparameter tuning via Bayesian optimization or neural architecture search.

### Data analysis and communication module

The data analysis and communication module is implemented as a lightweight embedded routine running on a low-power processor. It performs real-time EEG feature extraction and seizure prediction using the trained MCSV-CNN model. Predicted events are then communicated via Bluetooth to a mobile device or caregiver interface. The results of the SP were calculated to evaluate the efficacy of the suggested framework accurately. For a particular patient who experienced *U* seizures, using a hold-out validation technique, the recorded EEG signals were divided into training and testing sets, allotting 80% of the data for training and 20% for testing. In order to give the model enough data to identify broad trends and maintain a separate set for objective assessment, this split was selected. Furthermore, in order to increase the performance measures’ resilience, the split was carried out again using many other random seeds, and the average performance was published. This methodology ensures that the efficacy of the model is not attributable to a particular data configuration and aids in assessing its applicability to further data.

In this study, the CNN was trained using the EEG dataset, which included labels for the PIL stage occurring 5 minutes prior to a seizure. The rate of error of the validation dataset was computed after each training epoch to confirm whether the CNN overfitted the training dataset. The training process was halted when either the maximum number of iterations was reached or the validation error rate increased over ten consecutive training epochs. In this research work, it was possible to terminate the training process after around 150 iterations. After completing the CNN training, the PIL and IIL recordings from the clinical EEG signals were examined using the pre-trained CNN model. Calculation of evaluation measures like ACC, FPR, and SEN followed. The SPA-IoT model was set up on a Raspberry Pi 4 Model 4B. This device features a modern 64-bit quad-core processor that operates at 1.5 GHz and includes a metal heat sink. It also offers dual-band wireless LAN across 2.4 GHz and 5 GHz bands, as well as BLE gigabit Ethernet. The model is equipped with 8GB of RAM, improved dual-band 802.11 b/g/n/ac wireless LAN, Bluetooth 5.0, and a much faster 300 Mbit/s Ethernet. In this research, the offline EEG signals were relayed to the Raspberry Pi 4 via the internet on a continuous basis.

The following criteria are employed in this study for assessing the effectiveness of the proposed technique in quantitative terms:

10 $$Sensitivity (Sen)=\frac{TP}{TP+FN}$$

11 $${Accuracy (Acc)= \frac{TP+TN}{TP+FN+TN+FP}}$$

12 $${False Prediction Rate (FPR)= \frac{FP}{Time Period}}$$

where TP represents the True Positive;

TN represents the True Negative;

FP is the False Positive; and

FN represents the False Negative.

The sensitivity of the MCSV-CNN prediction model to pre-ictal data has made it possible to measure the classifier’s ability to gather this data. The sensitivity mentioned above refers to the particular capability of the proposed prediction framework to accurately recognize the pre-ictal EEG data as the pre-ictal stage, and this, in turn, is a measure of the ability of the classifier to identify the non-pre-ictal information. Accuracy refers to the prediction model’s potential to predict seizures accurately. The algorithm proposed in this work was tried out on epileptic data of a hundred patients who had suffered from more than a hundred seizures in both the TUH-EEG and the clinical EEG datasets.

In addition, for each patient, a separate seizure prediction model was trained and tested using their own EEG data. Performance metrics were calculated per patient, and results were aggregated to compute overall averages. This approach supports personalized seizure prediction, which is more realistic for clinical deployment scenarios.

#### System deployment

This is deployed on the server powered by Raspberry Pi 4, which can be reached by web and mobile users with the host’s IP address. In the event of any login request sent by a user, the central server itself can interact with many databases, both online and offline. Users can also use their local databases for offline operations or subscribe to certain databases for online procedures. The hosting server provides all the online data services. In this work, we connected to the TUH-EEG database, which contained data from a hundred patients. In future work, we will extend our system by connecting it to more free and paid databases. It has been implemented on a Raspberry Pi hosting server with log-in protocols and database management. It provides access to EEG data analysis and processing tools like MCSV-CNN. This server enables raw EEG data preprocessing, provides analytical tools for various research purposes, and offers classification and prediction models that are particularly helpful in seizure analysis. These are the services provided using cloud-based solutions. For this purpose, Azure has been selected as the main cloud platform. The integration of Python and YAML scripts helps manage these services effectively. Security is of major concern; handled within a private cloud, it ensures protection for user data and database integrity. JFROG does storage management. Additionally, edge computing handles IoT devices, which also have their separate sets of security and privacy vulnerabilities. It not only speeds up data handling and decision-making but also ensures data backup and secure transmission to data centers for further analysis and prolonged storage. Pre-deployment encryption and security measures are applied in a stringent way to keep the network environment safe for IoT devices and ensure safe data interaction and sharing.

## Results

In the current study, we analyze seizures predicted by the algorithm developed herein and confirmed by certified epileptologists. Our investigation makes use of a SP characteristic technique and utilizes specific performance metrics as delineated by the referenced indices  [28]. The focus of our analysis is the SP horizon, which is the time between when a seizure warning is issued and when the seizure actually begins. SOP stands for seizure occurrence period, which is a 10-minute window anticipated for the seizure onset. In the methodology, SPH is assumed to be 5 minutes. Predictive accuracy and early detection are balanced by the pre-ictal window selection. Although these decisions are consistent with previous research and provide enough time for behavioral reactions, they might reduce sensitivity to seizures involving more gradual or prolonged pre-ictal transitions  [28]. Additionally, potentially helpful near-miss predictions could be penalized by the stringent SOP cutoff. To better represent the variation in seizure onset patterns and enhance clinical value, future research should include dynamic or patient-specific pre-ictal modeling and graded SOP evaluation.

An SP is considered accurate if the seizure onset takes place within the SOP and not during the SPH. An accurate prediction is recorded as TP. On the other hand, when a seizure happens but is not predicted within the time frame of the SOP, it is labeled as an FN. Furthermore, not all alarms match real seizure events; if there is an alarm but no seizure in the SOP, it counts as a false positive. Such less-than-perfect predictions are bound to occur and make this system quite realistic, considering SP is a tough problem in reality.

**Fig. 5:**
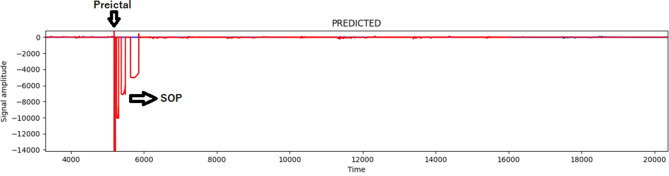
Result of SP by capturing the PIL stage in the EEG signal. Outcome of prediction achieved by detecting early EEG patterns during the PIL stage, enabling timely intervention before seizure onset

Figure 5 depicts the result of SPs. Fig. 5 shows that the MCSV-CNN model accurately predicted that the SOP phase came before the PIL phase. This proves that the method suggested in this work is ideal for predicting seizures accurately and with FPR.

The sensitivity of the MCSV-CNN prediction model to the PIL data enables the measurement of the classifier’s ability to gather this data. The specificity mentioned above refers to the particular capability of the proposed prediction framework to recognize the PIL EEG data as the PIL stage accurately, and this, in turn, indicates how well the classifier can recognize the non-PIL information. ACC is a reference to the potential of the prediction model to predict seizures accurately. The suggested scheme is applied to the epileptic data of a hundred patients who had suffered from more than a hundred seizures in both the TUH-EEG and clinical EEG datasets. Table 2 enumerates the results of the SPs made by the method in this study on all the patients. The average performance measures reported here are computed by combining evaluations on both the standard and clinical EEG datasets. However, to ensure clarity and transparency, we emphasize that the model was trained and validated separately on each dataset - no inter-dataset mixing occurred during training.

**Table 2:** Performance assessment of SP results

Dataset	Purpose	No.of signals	SEN	ACC	FPR
			$\%$	$\%$	(/h)
TUH-EEG	Training	100	99	99.8	0.09
Clinical	Testing	5	97.6	99.2	0
Average	-	-	98.3	99.5	0.045

Although the results report average performance metrics, the system was designed for patient-specific seizure prediction. That is, a separate model was trained and evaluated for each patient using only their own EEG recordings. The final performance metrics (ACC, SEN, FPR) were then averaged across all patients to provide an overall assessment. This individualized modeling approach aligns with previous work, acknowledging the high variability between patients in seizure patterns. No single patient was selected to represent performance. No data mixing across patients was done.

The average SEN of the SP for each of the one hundred patients was 98.3%. The average FPR was 0.045 per hour. The training and testing of the model took 150 epochs, achieving a testing ACC of 0.9898%. Up until 84 epochs, the percentage of the training ACC of the model was almost 0.9%. Beyond that, there was a small rise in the ACC of around 0.02%. It was observed that from 105 epochs to 150 epochs, no additional improvement was noticed in the ACC. Beyond training for 150 epochs, the development loss of the algorithm increased slightly, and there was a small decrease in its training loss. Still, it nearly leveled off, indicating that, most likely, the model was under-fitting the data. The AUC is a significant curve for measuring the prediction model. In this research, the AUC was computed to verify how the suggested SP model performed. A value that is as close to 1 as possible signifies a better performance of the model. The prediction model suggested in this work obtained an AUC of 0.9994, which is far greater than any of the recent advanced models  [22–25]. The dataset was partitioned on a per-patient basis using a leave-one-seizure-out cross-validation strategy. Specifically, for each patient, all but one seizure instance were used for training, and the held-out seizure formed the test set. This approach was repeated iteratively for all seizures, and average metrics were reported. To test generalizability, an additional cross-subject evaluation was performed in which data from all but one patient were used for training, and the model was tested on the unseen patient. Data balancing was ensured across the PIL and IIL classes in both training and test sets. All segments were padded or truncated to fixed lengths before input into the model.

The outcome of the experiments conducted in this study reveals that the SPA that is based on the MCSV-CNN can obtain an FPR of 0.045 per hour. This number is higher than that achieved by prediction techniques currently in use  [22–25]. Experimentation has revealed that the proposed technique performs better when there are high positive rates with lower false alarms. The conclusion that can be derived is that the proposed approach is able to predict seizures for epileptic patients effectively.

### System deployment

Wearable integration and real-time performance are key design considerations for the suggested seizure prediction system. To guarantee continuous monitoring, EEG data are obtained via a multi-channel wearable sensor and processed in overlapping windows. Important features are effectively extracted without requiring extensive preprocessing. Fast DWT is used in wavelet-based extensions to acquire multiscale representations with low latency. These characteristics are loaded into a low-resource platform-optimized lightweight MCSV-CNN, which achieves inference on embedded hardware in 30–50 ms per window. Real-time operation with minimum processing overhead and excellent temporal resolution is made possible by this approach.

**Fig. 6:**
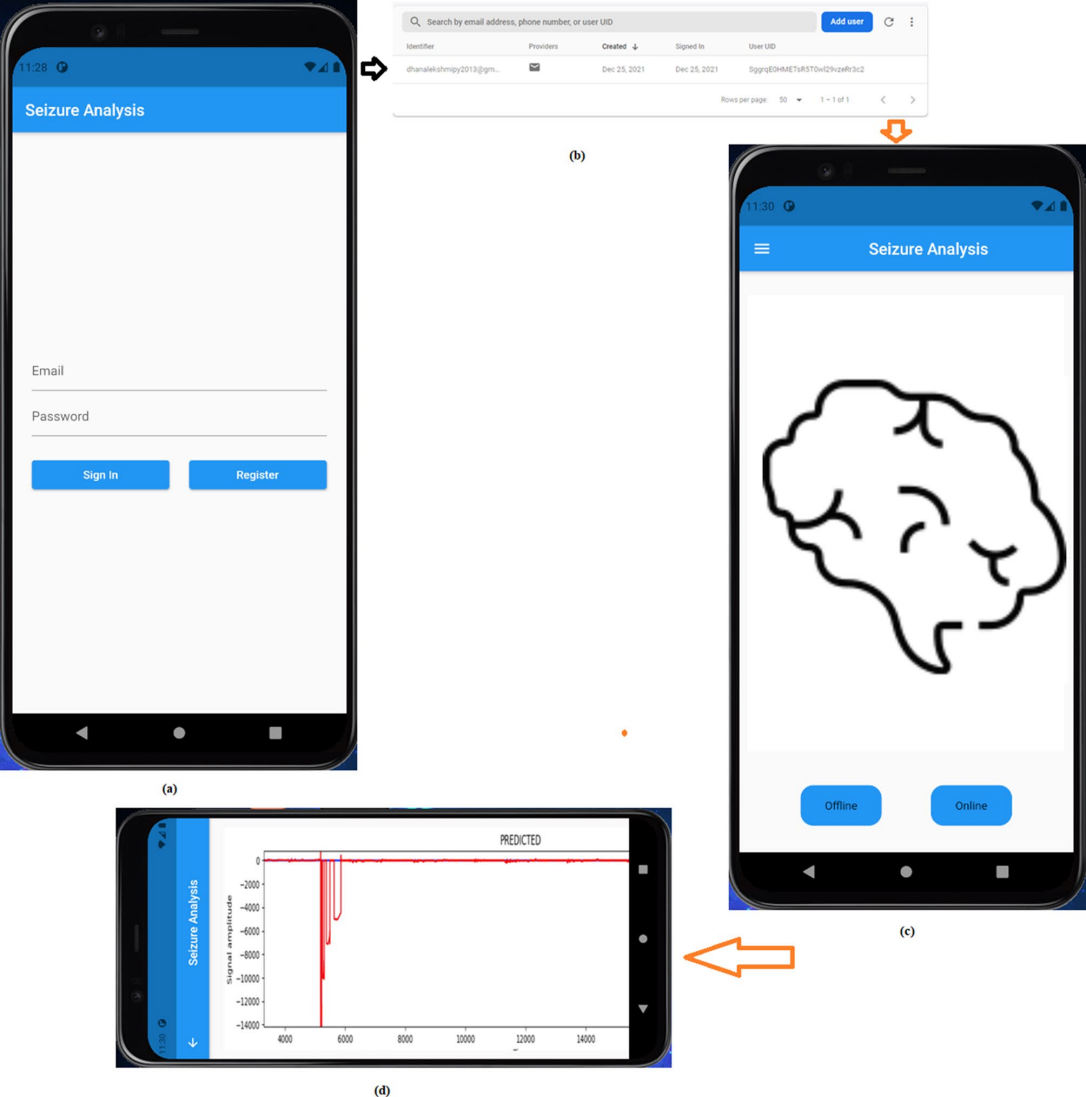
Result of SP by capturing the PIL stage in the EEG signal. Prediction outcome is derived by analyzing EEG signals during the PIL stage to anticipate seizures before onset

Figure 6 shows the predicted output using the seizure analysis application (App). The average SPA latency reported in this study is 0.93 seconds. This latency accounts for the preprocessing, extraction of high-frequency oscillations (HFO) from the seizure signal, and the use of a CNN for segment-by-segment prediction. The energy consumption for the transmission of the EEG data is 590 milliamps (mA). It is verified that the CNN model consumes less power for computation than other methods, such as support vector machine, k-nearest neighbor, and K-means clustering. This is because the Raspberry Pi’s CPU is completely ( > 90%) occupied for processing an EEG segment throughout the prediction phase. Hence, the proposed technique normalizes energy consumption in comparison to other methods.

### Ablation study

To better understand the contribution of individual components of the MCSV-CNN architecture, we conducted an ablation study by systematically removing or modifying key modules:

i. **Without Multiresolution Filtering**: Replacement of multiresolution input with raw EEG degraded the performance by 94.1%, confirming the importance of subband representations;
ii. **Without Sub-band Voting**: Aggregating outputs through simple averaging, instead of majority voting, resulted in a drop in sensitivity and increased FP; and
iii. **Shallower CNN**: Reducing convolutional layers led to poorer generalization in clinical datasets.

These results highlight that both the multiresolution decomposition and the structured subband voting mechanism are key to the robustness and accuracy of MCSV-CNN [Table 3].

**Table 3:** Ablation study on key MCSV-CNN components

Model Variant	SEN (%)	FPR/h	AUC
Full MCSV-CNN	98.3	0.045	99.94
Without multiresolution filtering	94.1	0.14	93.0
Without sub-band voting	91.5	0.29	91.0
Shallower CNN (2 conv layers)	89.1	0.34	88.6

## Discussion

### Results of seizure prediction for various frequency bands of EEG

MCSV is mainly found in the higher frequency bands in the case of IIL recordings. However, in the case of PIL recordings, the distribution of the MCSV is even in both the low and the high-frequency bands. To explore the impact of different frequency bands on SP results, features from various EEG bands were incorporated into the CNN to aid in seizure prediction. Once MCSV analyzes the EEG signals, a feature map that fits in a 0.1 Hz frequency resolution is fed as input to the CNN. The size of such a feature map was 21 ×the number of segments that contained the specific frequency components like *δ* (0–4 Hz), *θ* (4–8 Hz), *α* (8–13 Hz), *β* (13–30 Hz), $\gamma-1$ (30–70 Hz), $\gamma-2$ (70–128 Hz), and *fb* respectively. The average SEN of SP of seven frequency bands was 71.23%, 82.3%, 85.8%, 85.5%, 87.2%, 89.9%, and 97.7%, respectively. The average ACC was 80.2%, 86.6%, 87.3%, 82.3%, 94.3%, 94.9%, and 98.9% respectively. However, the ACC outcome of SP making use of the entire frequency band of the signal was significantly higher in comparison to just the six bands of the EEG signal. It has been observed that ES is associated not only with low-frequency signals but they are also with high-frequency areas of the signal  [28–30]. Given that each frequency band of the incoming EEG signal contains varying yet useful information that can be utilized in SP, integrating these features provides the CNN model with additional MCSV characteristics to categorize into IIL and PIL recordings. Therefore, the suggested technique significantly enhances the outcome of SP.

### Inequality analysis of PIL data

In the EEG data received from epilepsy patients, PIL epileptic zones are normally much shorter than IIL epileptic areas. As observed by the outcome of the experiments in this study, it was observed that the ratio of the IIL area to the PIL area is very high at 15:1. The imbalance in the data made the data unsuitable for training the CNN model due to the inadequacy in learning for samples of the minority class (PIL zones). To overcome the hurdles arising from class imbalance, data resampling techniques were utilized  [29, 30]. Out of the currently available methods, three techniques were selected, namely, random oversampling (ROS), borderline synthetic minority oversampling technique (bSMOTE), and random undersampling (RUS)  [31].

For ROS, the average Acc was 99.5%, and the SEN was 98%. The bSMOTE achieved an average Acc of 97.1% and an average SEN of 92%. RUS attained an average Acc of 89.1% and a SEN of 88%. It was observed that there were no major variations in the Acc values between ROS and bSMOTE in relation to SP results. These observations have led to the conclusion that using DL to overcome class imbalance issues in seizure prediction would make a sampling method like ROS a more convenient and easier technique compared to other methods, such as bSMOTE and RUS.

### Seizure behavior in scalp

**Fig. 7:**
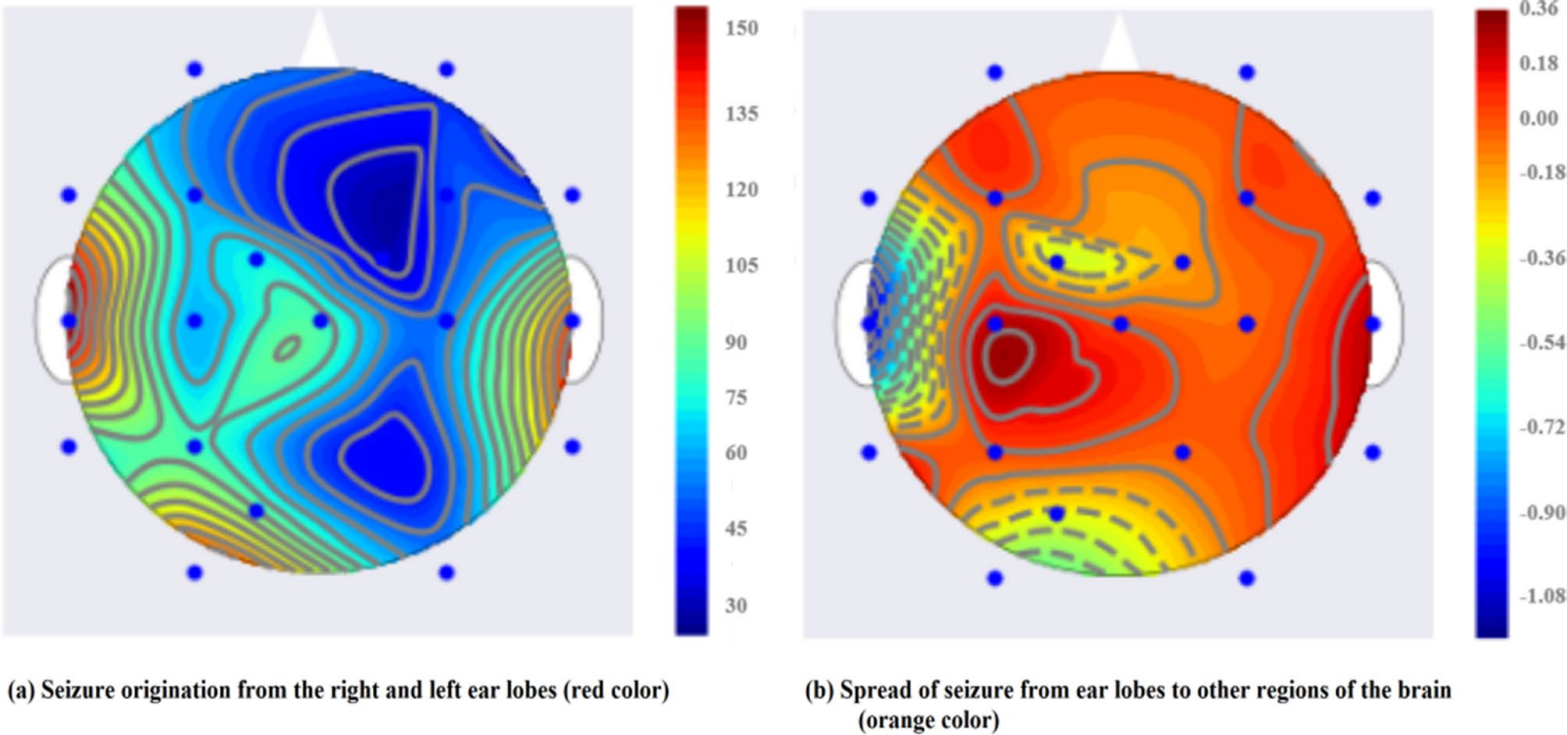
Topographic representation of the various IIL stages. This represents the illustration of the various phases of the IIL stage, highlighting spatial EEG dynamics between seizure events

The EEG topographic representation of the spread of spectrum in various frequency bands and sub-bands throughout the PIL and IIL phases is highlighted in Fig. 7. Important details regarding the structure and operation of the brain are learned from topographic organization. More significant values in the ictal state are indicated by red, while an ictal-free condition is indicated by blue. The spread of seizure activity (IIL state) in the relevant brain lobes is represented by the color orange. It is noted that the PIL stage, preceded by the IIL phase, is precisely captured by the proposed MCSV-CNN model. Fig. 7 demonstrates that the brain’s left and right ear lobes are sending out powerful signals. These signals are enhanced by the gamma and full frequency bands used in the MCSV-CNN technique. Subsequently, Fig. 7 shows a clear indication of seizure spread from the ear lobes to other brain regions following the seizure onset. The presented results are cross-verified with the data provided in the annotation  [22].

### Runtime and computational requirements

To assess the feasibility of real-time deployment, the proposed MCSV-CNN model was implemented using TensorFlow and tested on a system with an Intel i7 CPU (2.9 GHz), 16 GB RAM, and an NVIDIA GTX 1660 GPU. The average inference time per EEG window (5 seconds) was approximately 32 ms, well within the constraints of real-time seizure prediction. The model contains approximately 1.2 million parameters and requires 4.7 MB of storage, making it lightweight enough for integration into edge devices. Memory footprint and latency were further reduced by quantizing weights and pruning redundant filters after training, without a significant drop in accuracy (less than 1.5%). Preliminary tests on a Raspberry Pi 4B (ARM Cortex-A72, 4 GB RAM) showed that inference could still be performed under 100 ms per window, suggesting compatibility with low-power embedded systems. These characteristics support the scalability of the MCSV-CNN for continuous monitoring in resource-constrained wearable or IoT-based environments, provided efficient model deployment frameworks such as TensorFlow.

### Comparative analysis

This section discusses different SP methods that check the efficacy of the proposed MCSV-CNN-based approach. Table 4 presents a comparison between different methods for SP. Various works confirm the significance of the feature based on power spectra and the usage of DL models for SP. Both are decisive factors in a correct and rapid seizure diagnosis. The proposed technique from this paper has the highest performance in predicting seizures for all subjects, hence setting the highest benchmark performance. The MCSV-CNN method is superior to all others with respect to its ACC, SEN, and FPR. This is justified by two major principles: first, the multichannel EEG data used in the model captures holistic seizure-related information, and second, the capability of the MCSV-CNN model to detect HFOs effectively, which are important biomarkers of seizure activities. Comparing the proposed MCSV-CNN method with other techniques is challenging due to differences in channel-segment selection, specific features used, and classifiers employed in each method. However, from the literature review, it can be inferred that the MCSV-CNN technique outperforms others in performance values on all EEG channel segments, reflecting its robustness and efficiency for SP.

**Table 4:** Comparative analysis of the suggested SPA-IoT technique

Author	Method used	Performance analysis
		$$Sen/Acc/AUC in \%$$
		$$FPR in /hr.$$
Gao et al. [32]	Multi-scale features with weighted fusion based CNN	SEN-93.3,
		FPR-0.007
Xu et al. [33]	Preictal artificial signal synthesis algorithm	SEN-NS
	based on a GAN	ACC:78
Jemal et al. [34]	Interpretable DL classifier	SEN-94.96,
		FPR-0.096
Zhao et al. [35]	Adder network and supervised contrastive learning (AddNet-SCL)	SEN-89.1- 94.9,
		FPR-0.120–0.077,
		AUC: 83.1–94.2
Lopes et al. [36]	Ensemble deep neural network based on the	SEN-92.84,
	combination of time-series, PSDs, and topoplots	
Albaqami et al. [37]	WaveNet based Long Short Term Memory	SEN-97.09,
		ACC:97.45
Yang et al. [38]	Multilevel temporal spectral feature	
	extraction network	ACC:85.7
Wei and Mooney [39]	Twenty features based on Time and	
	frequency domain features	ACC:98.95
Bidgoli et al. [40]	Generative Adversarial Networks	ACC:71.27
		AUC:72
The proposed technique	SPA system using	SEN-98.3,
	Multiresolution critical spectral verge (MCSV)	AUC: 99.94,
	and CNN technique	ACC:99.5,
		FPR-0.045

### Limitations and future work

This study suggests employing the MCSV-CNN scheme for SP. The primary difficulty in SP lies in choosing the right algorithm to process and evaluate the EEG data and correctly identify the various epileptic states. Furthermore, our prediction is substantially hampered by the fact that a sizable fraction of the PIL EEG signal remains highly identical across periods in the same patient. Through the capture of HFOs in EEG data, this research concentrated on the PIL phase. In addition, the CNN model predicts seizure activity based on segment-wise multichannel HFO extraction. Nevertheless, the outcome will be significantly improved if the DL algorithm makes use of an adaptive approach for seizure details. DL model architecture continues to be hampered by IoT device asset limitations. Efficacy and time efficiency are two major issues in the design of DL in actual IoT systems. The future of the proposed approach lies in being able to attain cross-patient prediction effectively. That would be a major enhancement for this work. Moreover, the proposed system has been deployed using private data and a private API in a confined space. However, considering that numerous connected things exist, future systems will be implemented in open settings, referred to as software environments, with a dizzying array of services offered.

In addition, while the current evaluation benchmarks demonstrate that the proposed MCSV-CNN outperforms several traditional DL approaches, we acknowledge the increasing relevance of more recent architectures, such as graph neural networks (GNNs) and transformer-based models, particularly for EEGs spatial-temporal structure. Future work will involve benchmarking MCSV-CNN against these state-of-the-art models to provide a more comprehensive performance comparison, especially in wearable IoT scenarios where attention mechanisms and graph representations can capture inter-electrode dependencies more explicitly  [41],   [42].

Although there is potential for real-time clinical and ambulatory applications of the suggested IoT-based seizure prediction system, there are a number of practical and ethical issues with its implementation. The most prevalent risks include false negatives, which result in missed seizure warnings and potential injury, and false positives, which cause needless worry or disruption of lifestyle. The system is assistive, not deterministic, and ought to be avoided in place of clinical judgment; this must be made apparent.

While the proposed MCSV-CNN framework demonstrates promising preliminary results, it is important to note that the size and diversity of the datasets used limit the current study’s validation. Several statements regarding model generalizability, robustness, and clinical applicability remain to be confirmed through larger-scale, multi-center studies and prospective real-world testing. Future work will focus on expanding validation to include more heterogeneous patient populations, rigorous ablation studies to isolate key architectural contributions, and deployment trials in realistic IoT environments to solidify the claims made.

Lastly, as the system is a decision-support tool that may have medical ramifications, regulations should govern it, undergo clinical studies, and be continuously monitored after implementation. Safe and ethical incorporation into epilepsy care will depend on ensuring adherence to medical device laws and including stakeholders, such as patients, neurologists, and caregivers.

## Conclusion

A monitoring system SPA and IoT-enabled SP based on the suggested MCSV-CNN architecture were presented and assessed in this work. When compared to other approaches, the method showed a lower FPR, better AUC scores, and good precision in detecting pre-ictal regions in EEG recordings. According to experimental findings, seizure-related patterns have been observed throughout the entire EEG spectrum, especially in the high-frequency components. Additionally, the system demonstrated resilience in noisy and real-time environments, suggesting that it might be used practically in wearable and clinical monitoring scenarios.

## Data Availability

The TUH-EEG datasets used in this paper is publicly available at https://isip.piconepress.com/projects/nedc/html/tuh_eeg/. The clinical data set is available upon reasonable request https://isip.piconepress.com/projects/nedc/html/tuh_eeg/.
